# Metabolic Syndrome and Sleep Disturbances in Brain Disorders: Mechanisms, Implications, and Therapeutic Pathways

**DOI:** 10.1002/alz70855_106291

**Published:** 2025-12-24

**Authors:** Aakanksha Verma

**Affiliations:** ^1^ Maharishi Markandeshwar Deemed to be University, Ambala, Haryana, India

## Abstract

**Background:**

The intricate relationship between gut microbiota, sleep regulation, and mental health is a complex phenomenon that warrants further investigation. Research has shown that the gut microbiota plays a crucial role in modulating sleep patterns, mental health, and metabolic disorders.

**Methods:**

This compilation of research includes a systematic review of the impact of gut microbiota on sleep regulation, particularly in conditions like diabetes and metabolic syndrome. Additionally, a study examines the sedative properties of Naoling Pian (NLP), a traditional Chinese medication, in treating insomnia. A review of the literature also explores the connection between the gut microbiota and psychiatric disorders.

**Results:**

The systematic review reveals that the gut microbiota composition significantly affects sleep regulation, and that dietary choices can modulate microbial composition and metabolites, impacting sleep and metabolic syndrome. The study on NLP demonstrates its therapeutic benefits in treating insomnia, with effects similar to those of diazepam. The review of psychiatric disorders highlights the correlation between gut microbiota, sleep quality, and mental health, with specific bacteria linked to improved sleep quality in bipolar disorder.

**Conclusion:**

These findings underscore the complex interplay between gut microbiota, sleep regulation, and mental health, and highlight potential therapeutic pathways for treating metabolic disorders, insomnia, and psychiatric disorders.

Keywords: Gut microbiota, Insomnia, Mental health, Metabolic syndrome, Sleep regulation.

